# Prevalence of Hearing, Vision and Cognitive Impairment and Impact in Older Adults in Home Care: A Study Protocol

**DOI:** 10.1111/hex.70613

**Published:** 2026-04-15

**Authors:** Melinda Toomey, Lana Wilson, Helen Gurteen, Bronwyn Franco, Rebecca Bennett, Dayna Cenin, Najwan El‐Saifi, Melanie Ferguson, Yuanyuan Gu, Chyrisse Heine, Lisa Keay, Sheela Kumaran, Sabrina Lenzen, Iracema Leroi, Judy A. Lowthian, Carly Meyer, Leander Mitchell, John Newall, Nancy A. Pachana, Marianne Piano, Smriti Raichand, Emma Scanlan, Hamid Sohrabi, Piers Dawes

**Affiliations:** ^1^ Centre for Hearing Research, School of Health and Rehabilitation Sciences University of Queensland Brisbane Australia; ^2^ National Acoustic Laboratories Macquarie University Sydney Australia; ^3^ Brightwater Research Centre Perth Australia; ^4^ School of Allied Health Curtin University Perth Australia; ^5^ Macquarie University Centre for the Health Economy, Macquarie Business School and Australian Institute of Health Innovation Macquarie University Sydney Australia; ^6^ Institute of Health and Wellbeing Federation University Australia Ballarat Australia; ^7^ School of Optometry and Vision Science UNSW Sydney Sydney Australia; ^8^ Centre for the Business and Economics of Health The University of Queensland Brisbane Australia; ^9^ Department of Psychiatry, School of Medicine, Trinity College Dublin Global Brain Health Institute Dublin Ireland; ^10^ School of Public Health and Preventive Medicine Monash University Melbourne Australia; ^11^ Bolton Clarke Research Institute Brisbane Australia; ^12^ School of Psychology The University of Queensland Brisbane Australia; ^13^ Department of Linguistics, Faculty of Medicine, Health and Human Sciences, The Australian Hearing Hub Macquarie University Sydney Australia; ^14^ Department of Optometry and Vision Sciences, Melbourne School of Health Sciences University of Melbourne Melbourne Australia; ^15^ Australian College of Optometry National Vision Research Institute Melbourne Australia; ^16^ Hearing Australia Sydney Australia; ^17^ Centre for Healthy Ageing, Health Futures Institute Murdoch University Perth Australia; ^18^ Manchester Centre for Audiology and Deafness University of Manchester Manchester UK

**Keywords:** cognitive impairment, dementia, hearing impairment, home care services, older adults, quality of life, vision impairment

## Abstract

**Background:**

Hearing, vision and cognitive impairments are common yet frequently underrecognized among older adults. Although these impairments affect quality of life, functional independence and psychological well‐being, there are no published data on the prevalence and consequences of these impairments in relation to Australian home care populations. This protocol outlines a cross‐sectional investigation into the prevalence of hearing, vision and cognitive impairments and their associations with quality of life, functional ability and psychosocial well‐being among older Australians receiving home care services.

**Methods:**

A total of 369 participants aged 65 years and older will be recruited from home care services across Australia. Standardized assessment tools will be used to assess hearing, vision and cognitive function, quality of life, daily living activities, mental health and social participation. Multi‐variable regression models will explore the impact of sensory and cognitive impairments on health and well‐being outcomes.

**Discussion:**

With ageing populations, it is increasingly important to support older people to live independently in their own homes rather than needing to move into residential aged care. This study will facilitate understanding of the prevalence and impact of sensory and cognitive impairments among the older Australian home care population. Findings may inform strategies to support health ageing in place, including service planning, care coordination and workforce training.

**Patient or Public Contribution:**

Older adults receiving home care services and individuals with lived experience of sensory and cognitive impairments contributed to the study design. A Patient and Public Involvement advisory group and a stakeholder steering group will guide study implementation.

## 1 Introduction

In Australia, there is a strong societal and policy emphasis on supporting older adults to ‘age in place’, that is, to remain in their own homes and communities for as long as possible, rather than transitioning to residential aged care [1]. This preference is reflected in the growing demand for home care services [2]. To meet this demand, the Australian Government funds home support services, which provide a range of services delivered by professional caregivers, including nursing, allied health, personal care, domestic assistance and social support [3]. These services are designed to help older adults living in the community maintain independence and quality of life. Despite these services, health‐related factors, including hearing, vision and cognitive impairments, can compromise the ability of some older adults to age in place, particularly when hearing, vision and cognitive impairments go unrecognized, are suboptimally managed, or occur in combination. While many individuals with well‐managed hearing or vision impairment can live independently with minimal support, cognitive impairment may reduce the ability to compensate for hearing/vision loss, manage assistive devices, navigate hearing/vision services and maintain safety [4]. Comorbid hearing/vision impairment and cognitive impairment is therefore especially relevant to functional decline, increased care needs and transition to long‐term care [5, 6]. Hearing/vision and cognitive impairments are common among older people and are associated with reduced quality of life, increased functional limitations and higher care needs [6, 7]. Yet the prevalence and impact of hearing/vision or cognitive impairments in the Australian home care population remain poorly understood.

Population‐based audiometric testing of Australians found that hearing impairment (defined as four‐frequency average hearing level [4FAHL] worse than 25 decibels [dB] in the better ear) affected approximately 16.3% of individuals aged over 50 years and 62.8% of those aged over 70 years [8]. Although hearing loss is common and affects communication in daily life, only around 15% of Australians with hearing loss use hearing aids [9], with barriers including cost, stigma, device‐related issues and limited access to services [10].

Vision impairment, defined as visual acuity worse than 6/12, affects 6.6% of Australians aged 50 years and older [11]. Long‐term vision disorders (e.g., refractive error, cataracts, macular degeneration and glaucoma) affect up to 93% of Australians aged 65 years and over [12]. The high prevalence of long‐term vision disorders in older adults is concerning, as these eye conditions (e.g., macular degeneration, glaucoma) require timely detection, treatment and ongoing care to prevent or slow down the progression of vision impairment. Dementia is also increasingly prevalent, with an estimated 84 people with dementia per 1000 Australians aged 65 years and older in 2023 [13]. In 2022, 54% of older adults living in residential aged‐care in Australia had dementia [14], but there are no published data about the prevalence in Australian home care settings that we are aware of.

Internationally, studies have highlighted high levels of hearing, vision and cognitive impairment among older adults receiving home care. Recent research in Canada used the InterRAI Home Care assessment to estimate prevalence levels. Hearing impairment affected approximately 25%–29% of home care recipients and vision impairment 12%–14%, while 15%–21% had dual sensory impairment [15]. In this Canadian study, hearing and vision impairment were determined using clinician‐rated resident assessment instrument for home care (RAI‐HC) (www.interrai.org) items that assess functional hearing and vision based on interview, observation and caregiver input. Ratings of hearing and vision were based on functioning while individuals were using their usual corrective devices (i.e., glasses and hearing aids, if worn). Impairment was defined as the presence of at least mild functional difficulty in the relevant sensory domain. A Norwegian study of home care recipients aged 80 years and older, found that 47% had moderate hearing impairment (defined as 4FAHL between 40 and 60 dB HL) and 40% were visually impaired (defined as visual acuity of ≤ 0.4 logarithm of minimum angle of resolution [logMAR]), with an additional 41% and 56% having slight hearing (4FAHL between 25 and 40 dB HL) and visual impairments (visual acuity between 0.4 and 0.5 logMAR), respectively [16]. In the UK‐based PrOVIDe study, which examined vision in people with dementia aged 60 to 89 years, the prevalence of mild vision impairment (presenting visual acuity worse than 6/12) among those living at home was reported at 21.8%, and moderate or worse vision impairment (presenting visual acuity worse than 6/18) was 10.6% [17]. In the Irish home care population, the prevalence of dementia and cognitive impairment was estimated to be 37.1% and 8.7%, respectively, based on documented clinical diagnoses of dementia or cognitive decline affecting independent living or suspected cognitive impairment identified using validated cognitive screening tools [18]. Analysis of Canadian interRAI data estimated the prevalence of cognitive impairment to be 23.2% in home care clients using the interRAI cognitive performance scale [6].

The current study was informed by the Maryland Assisted Living Study, which examined the prevalence of dementia and psychiatric disorders and their impact on functional impairment in assisted living populations in the USA [19, 20]. Research highlights the importance of considering multi‐morbidity, particularly the co‐occurrence of sensory and cognitive impairment, in shaping health and functional outcomes [21]. Evidence from the SENSE‐Cog programme has demonstrated that hearing and vision impairments are associated with accelerated cognitive decline compared with those with no sensory impairment [22], that comorbid hearing, vision and cognitive impairments lead to unmet hearing/vision support care needs [4, 23] and are detrimental to quality of life [24]. Moreover, overlapping symptomology between hearing, vision and cognitive impairment, such as disorientation, communication difficulties and increased risk of falls, can complicate accurate diagnosis and discrimination of sensory versus cognitive problems [25]. Recognition of these complex interactions underscores the need to investigate sensory‐cognitive multi‐morbidity in older adults who receive home care services.

To supply an understanding of the prevalence of, and interactions between, sensory and cognitive impairments among older adults receiving home care services in Australia, we planned a cross‐sectional survey study. In addition to estimating prevalence, the study also entails modelling the impact of hearing, vision and cognitive impairments on quality of life, functional ability and psychosocial well‐being. Findings will inform healthcare delivery and policy to support ageing in place by addressing sensory and cognitive needs.

## 2 Methods

### 2.1 Study Design and Research Questions

This cross‐sectional study will address two research questions:

1. What is the prevalence of hearing, vision and cognitive impairments, both individually and in combination, among older adults receiving home care services in Australia?
2. What is the association of hearing, vision and cognitive impairments on the quality of life, functional ability and psychosocial well‐being of older adults receiving home care services in Australia?

**Figure 1:**
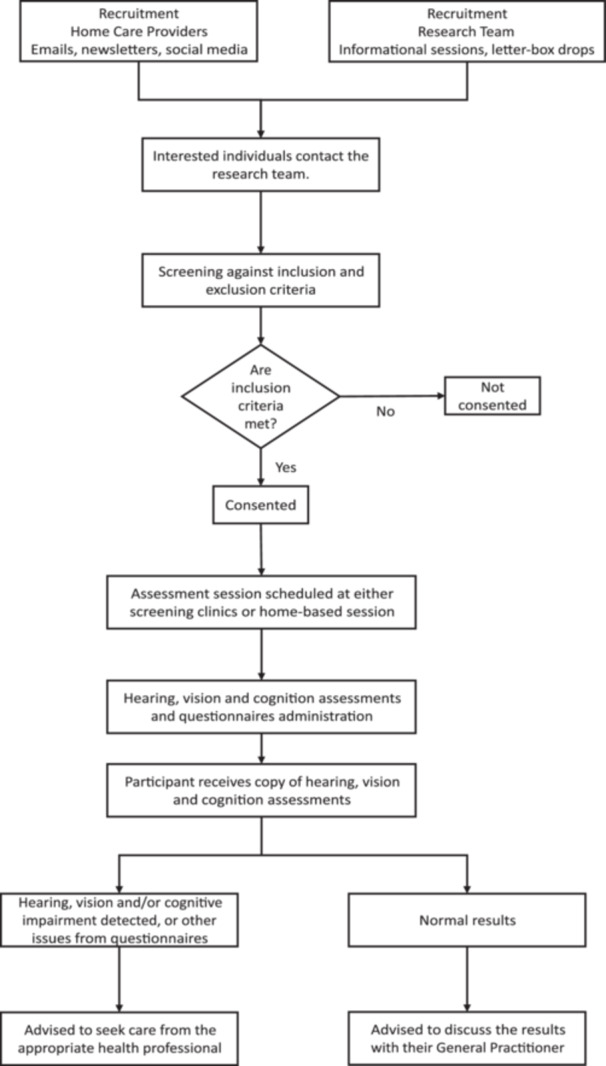
Study flowchart showing recruitment methods, screening, consent, assessments and pathways for dealing with normal and abnormal results.

Figure 1 provides a flowchart of the study. This protocol follows the Strengthening the Reporting of Observational Studies in Epidemiology (STROBE) statement (Supporting Information 1) [26].

### 2.2 Setting

This study will be conducted in Brisbane and surrounding urban areas in South‐East Queensland, Perth, Western Australia and regional locations across Queensland and Northern New South Wales. Assessment will take place in community rooms located within retirement villages, where multiple participants may be seen in a single day. For community‐dwelling older adults who are unable to attend these clinics, assessments will be conducted in their homes.

### 2.3 Participants and Sample Size

Participants will be included in the study if they are (i) aged 65 years or older, (ii) receiving home care services, (iii) able to provide consent and (iv) have adequate conversational English. The research team will informally assess conversational English during initial contact by determining whether the participant can understand simple instructions, respond to basic questions and engage in dialogue relevant to the study assessments. Individuals will be ineligible to participate if they have been hospitalized in the previous 2 weeks following acute illness, delirium or major infection. The planned sample size for the study is 369. Sample sizes were calculated for each impairment individually using the Scalex SP calculator [27], based on estimates of prevalence from international studies of home care populations with respect to hearing impairment (60%) [28], vision impairment (21.8%) [17] and dementia (67.7%) [19]. The required sample sizes were determined to be 369, 262 and 342, respectively, using a 95% confidence level and a ±5% margin of error. The final sample size meets or exceeds the calculated requirements.

### 2.4 Recruitment

To ensure a representative sample of home care recipients, participants will be recruited from a variety of home care providers. These organizations collectively reflect the diversity of the Australian home care sector in terms of provider type, size and service model. Three of the providers are large not‐for‐profit providers with national or multi‐regional reach, while the fourth offers a smaller, community‐based model. This mix captures variation in organizational scale, governance structures and funding arrangements (including public and private sources). Geographically, recruitment will span Southeast Queensland, regional Queensland, Northern New South Wales and Perth, Western Australia, enabling inclusion of participants from both metropolitan and regional settings and a broad range of socioeconomic levels. Recruitment through multiple home care providers operating across these regions reduces reliance on single‐site or single‐community sampling and increases socioeconomic diversity among participants. This recruitment approach enhances the generalizability of findings by reflecting the heterogeneity of home care provision across Australia.

Recruitment of older adults into research is inherently challenging, particularly for those with frailty, cognitive impairment or limited social support [29]. Thus, recruitment advertisements will be disseminated by our home care provider partners via email, newsletters, social media and direct contact from home care provider staff. Also, the research team will conduct information sessions at home care provider retirement villages and community centres in conjunction with letter‐box drops of written information about the study. Individuals can register their interest in participating online or contact the research team via phone or email. A research team member will screen participants against inclusion criteria and, if eligible, will schedule an assessment appointment.

### 2.5 Data Collection

Trained researchers will assess hearing, vision and cognition using standardized assessments, then administer a survey that collects demographic, quality of life, functional ability and physical and mental well‐being data. The assessment is expected to take 90 min for each participant. Participant‐centred adjustments include proxy completion of questionnaires for participants with dementia, support from a significant other during the assessment, regular breaks, or the option to complete on another day. All data will be entered into an online data collection platform (Qualtrics XM, Provo, UT, USA). Upon completion of the assessment, participants will receive a summary of their hearing, vision and cognitive assessment results (Supporting Information 2). Participants who fail the hearing, vision and/or cognition assessment or if concerns are identified from the questionnaires will be advised to seek care from the appropriate health professional, including an audiologist for hearing issues, an optometrist for vision issues and a general practitioner for cognitive concerns, depression or anxiety. Participants with normal results will be advised to discuss the results with their general practitioner and seek further support if needed.

### 2.6 Hearing, Vision and Cognitive Assessments

The definitions of hearing, vision and cognitive impairment that will be used in this study are outlined in Table 1.

**Table 1:** Definitions of hearing, vision and cognitive impairment with criteria for identification of impairment.

Impairment	Test	Criteria for impairment
Hearing	Pure tone thresholds	Four frequency average (500 Hz, 1 kHz, 2 kHz, 4 kHz) worse than 25 db HL
Vision	Visual acuity	Worse than 6/12 in either eye
	Contrast sensitivity	Worse than 70 Spaeth–Richman contrast sensitivity (SPARCS) score in either eye
	Central vision	Any distortion of the Amsler grid in either eye
	Visual fields	Any constriction of visual field in either eye
Cognition	Montreal Cognitive Assessment (MoCA)	Worse than 25 (out of 30)

#### 2.6.1 Hearing Assessments

Otoscopic examination using a Welch Allyn pocket LED otoscope (model number 22870‐BLK) (Welch Allyn Inc, Skaneateles Falls, NY, USA) will be conducted before the hearing assessment to identify wax occlusion or anatomical abnormalities. Participants found to have impacted wax occluding the ear canal will be referred to a health professional for removal and rescheduled to complete all assessments on another day. Hearing will be assessed unaided using the Hear X HearTest digital automated audiometer (HearX Group, Pretoria, South Africa), which measures pure tone thresholds [30]. Testing unaided reflects standard audiological practice and allows the use of standardized classifications of hearing impairment and compatibility with population‐based epidemiological studies of hearing impairment. Functional hearing impact in the everyday context will be captured through questionnaires (see section on questionnaires below). The HearTest assessment is administered on a Samsung tablet (Samsung C&T Corporation, Sunwon‐si, Republic of Korea) using calibrated Sennheiser HD 280 Pro headphones (Sennheiser V+V GmbH & Co, Wedemark, Germany). The testing protocol includes pure‐tone evaluation at 500, 1000, 2000, 4000 and 8000 Hz. The HearTest was selected because its accuracy and reliability compared with conventional audiometry have been established, and because the HearTest device is portable and allows automated data capture, which makes it well‐suited for community and home‐based research contexts [30].

#### 2.6.2 Vision Assessments

Vision will be assessed based on the presenting vision of the participant; those who use glasses will be tested while wearing them, and those who do not will be assessed unaided. This approach enables assessment of participants’ functional vision in conditions that reflect their typical daily experience. Each eye will be tested separately for distance visual acuity using the Peek Acuity Smartphone app [31], contrast sensitivity by Spaeth–Richman contrast sensitivity (SPARCS) [32], the Amsler grid to detect central vision disturbances [33] and confrontation testing using kinetic fingers for visual field assessment [33]. Visual acuity will be the primary measure to define vision impairment in this study. Contrast sensitivity will also be assessed as an alternative metric, given emerging evidence that it may identify functional vision deficits not captured by acuity alone [34]. Central vision will be evaluated using the Amsler grid, and visual fields will be assessed via confrontation with kinetic finger movements. While central vision and visual field tests will not be used to define vision impairment status, they are included to provide a more complete profile of visual function than visual acuity alone.

The Peek Acuity application will be administered on a smartphone that presents standard 5 × 5 grid ‘E’ optotypes in one of four orientations at the initial test distance of 2 m or 1m if required. The assessor remains unaware of the optotype being presented and whether the participant's response is correct, helping to minimize the potential for verbal or non‐verbal cues that could influence the outcome. Visual acuity is reported in LogMAR. For participants with low vision, Peek Acuity provides standardized digital alternatives to low‐vision assessments of counting fingers, hand movement and light perception. Smartphones used by the data collectors will be calibrated so that the test E's height and width are between 38 and 42 mm using the method described on the PEEK acuity site (https://peekvision.org/solutions/peek-acuity/peek-acuity-calibration/). PEEK Acuity was selected as it has been validated against standard chart‐based testing and offers a portable, scalable and accessible approach for use in community and clinical settings [31].

The internet‐based SPARCS test will be displayed on a portable 17.3‐inch external monitor (Arzopa, Guangdong, China). The SPARCS test evaluates contrast sensitivity across five distinct regions of the visual field (central, upper left, lower left, upper right and lower right quadrants), resulting in a log‐based score for each quadrant, which is then scaled out of 20. A maximum cumulative score of 100 indicates optimal contrast sensitivity. SPARCS was chosen because it allows assessment of both central and peripheral contrast sensitivity, providing a broader measure of visual function than traditional chart‐based methods, and is particularly sensitive to subtle visual deficits that may not be detected by acuity testing alone.

Central vision will be assessed using the Amsler Grid, a qualitative tool that evaluates the central 10° of the visual field. Participants will view the grid monocularly at a distance of 30 cm using their usual near correction if worn [33]. They will be asked to report any distortions, blurring or missing areas within the grid pattern, which may indicate macular or central retinal abnormalities. Visual fields will be assessed using confrontation testing with kinetic finger movements [33]. The participant and researcher will sit facing each other at approximately 1 m and at the same eye level. Each eye will be tested separately by comparing the participant's visual field to that of the examiner, using inward‐moving finger targets along multiple meridians. Participants will indicate when the moving target first becomes visible, allowing for a gross evaluation of peripheral vision.

#### 2.6.3 Cognitive Assessment

Cognitive function will be evaluated using the standard Montreal Cognitive Assessment (MoCA) tool [35]. Where appropriate, the hearing‐ and vision‐impaired versions of the MoCA will be used, based on the clinical judgement of the researcher assessing the participant [36, 37]. These adapted versions mitigate the impact of sensory loss by substituting items that rely heavily on auditory or visual input with alternative formats. For example, replacing verbal instructions with written prompts in the MoCA hearing impaired version and removing visual items in the MoCA‐vision‐impaired version [38]. Selection of the appropriate version will be guided by the participant's sensory profile. Use of the hearing‐impaired version will consider factors such as the degree of hearing loss, hearing aid use and observed communication ability of the participant during the assessment. The vision‐impaired version will be applied when participants demonstrate difficulty with visual components of the standard MoCA, or as informed by self‐reported vision history and assessment results.

#### 2.6.4 Demographic and Health Status

Sociodemographic information that will be collected includes age, gender, indigenous status, marital status, education, previous occupation and key aged care information, including package level, duration of home care and services received. Self‐reported health information that will be collected includes general health status, comorbidities, medication use, frequency of visits to their general practitioner and hearing, vision and cognitive status, as well as sensory device use (e.g., hearing aids, cochlear implants, glasses) (Supporting Information 3).

### 2.7 Questionnaires

Participants will be encouraged to use their usual hearing and vision devices during questionnaire administration to support communication and ensure optimal performance.

#### 2.7.1 Health‐Related Quality of Life

The health utilities index mark 3 (HUI3) is a 40‐item, interviewer‐administered instrument that assesses eight attributes of health—vision, hearing, speech, ambulation, dexterity, emotion, cognition and pain—over a 1‐week recall period [39]. It produces both multi‐attribute utility scores (range −0.36 to 1.00) and single‐attribute scores (range 0.00–1.00). The HUI3 has been validated across diverse populations, including people with hearing impairment and dementia [40, 41]. The proxy (interviewer)‐administered version of the HUI‐3 will be used for all participants. This version was selected to align with a parallel project in residential aged care, where most participants had cognitive impairment, allowing for shared licensing costs [42]. In this study, researchers will administer the HUI‐3 directly to participants. If a participant lacks the capacity to respond, a family member or significant other will be invited to complete the assessment on their behalf. Participants are not required to have a proxy present to be included in the study.

The quality of life—aged care consumer (QOL‐ACC) is a brief six‐item tool co‐developed with older adults receiving aged care services [43]. It captures the domains most valued by this group, including mobility, independence, emotional well‐being, pain management, social connections and activities. Scoring differentiates levels of quality of life from ‘very poor’ to ‘excellent’, and validation studies support its use in both people living with dementia and home care populations [44].

#### 2.7.2 Social Care‐Related Quality of Life

The adult social care outcomes toolkit (ASCOT‐SCT4) is a nine‐item self‐report instrument designed to measure outcomes specifically related to social care [45]. It is a multi‐attribute utility tool covering aspects such as control, personal safety and social participation. Scores range from −0.17 to 1.00, where higher values indicate better quality of life. The tool has demonstrated validity and reliability in populations with long‐term health conditions, including sensory impairments [45].

#### 2.7.3 Social Interaction

Social connectedness will be evaluated using three items adapted from the UK Biobank cohort study, which examines the frequency and quality of social interactions. Participants will be asked: (1) ‘How often do you visit friends or family or have them visit you?’; (2) ‘Which of the following leisure or social activities do you engage in once a week or more often?’; and (3) ‘How often are you able to confide in someone close to you?’ [46] Scoring will be applied such that one point will be assigned if participants reported fewer than one visit per month for Question 1, if they did not engage in any listed activities for Question 2 and if they reported being able to confide in someone less than once per month for Question 3. Higher scores indicate greater social isolation [47].

#### 2.7.4 Functional Ability

The instrumental activities of daily living (IADL) scale comprises eight items that assess independent living skills, including managing finances, transportation and medication [48]. Scores range from 0 (dependent) to 8 (independent).

#### 2.7.5 Hearing Function

The revised hearing handicap inventory for the elderly—screening version (HHIE‐S) is a 10‐item questionnaire measuring the emotional and social impacts of hearing loss [49]. Scores range from 0 (no handicap) to 40 (maximum handicap). The revised version has undergone psychometric re‐evaluation and demonstrates robust reliability and validity [49].

#### 2.7.6 Vision Function

The National Eye Institute Visual Function Questionnaire (NEI VFQ‐25) measures the self‐reported impact of visual impairment on daily functioning and quality of life across multiple domains. The 25‐item questionnaire includes subscales that assess global vision, near and distance vision activities, social functioning, role limitations, dependency, mental health, driving, peripheral and colour vision and ocular pain. Items are scored from 0 to 100, with higher scores representing better visual functioning [50]. It has been validated in both ophthalmic clinics and community‐based samples.

#### 2.7.7 Psychological Wellbeing

The geriatric anxiety inventory—short form (GAI‐SF) is a five‐item measure of anxiety in older adults [51]. Scores ≥ 3 indicate clinically significant anxiety. It has been validated against the longer GAI and is efficient for use in clinical and research contexts [51].

The geriatric depression scale—short form (GDS‐5) is a five‐item tool designed to screen for depressive symptoms in older adults [52]. A score of 2 or above suggests possible depression. It is widely validated and recommended for use in time‐limited settings [52].

### 2.8 Statistical Analysis

Statistical analyses will be performed using the Statistical Package for the Social Sciences (SPSS) version 29 (SPSS Inc., Chicago, IL, USA). Descriptive statistics will summarize participant characteristics, impairment assessment outcomes and health and well‐being measures. Prevalence estimates for hearing, vision and cognitive impairment, as well as their co‐occurrence, will be reported with 95% confidence intervals overall and by subgroups (age, sex, site, geographic location and home‐care package level). Associations between impairments (individually and jointly) and outcomes, including health‐related quality of life, social care–related quality of life, social isolation, functional ability and psychosocial measures, will be modelled using regression models appropriate to the outcome type (linear, logistic, ordinal or beta regression). All models will adjust for potential confounders, including age, sex, education, language, comorbidities and care package level. Effect modification by age, sex and site will also be explored. Missing data will be handled using multiple imputation under the assumption of missing at random (MAR). If data are missing not at random (MNAR), results may be biased; therefore, sensitivity analyses will be conducted to assess robustness. A value of *p* < 0.05 will be considered statistically significant.

### 2.9 Ethics, Data Management and Dissemination

The study received ethics approval from the University of Queensland Human Research Ethics Committee (HE002236), as well as governance approvals from each of the home care providers. Participants will receive study information and provide written consent, with the option to withdraw at any time. Accessible materials and proxy consent procedures are in place for individuals with dementia or limited capacity, including verbal agreement and witnessed consent where needed. In line with the National Health and Medical Research Council's Open Access Policy (2022) and FAIR Principles [53], and with participant consent, non‐identifiable data will be archived in The University of Queensland's Research Data Management System (UQRDM), a publicly accessible repository. Datasets and analysis scripts will be available upon reasonable request. Results will be disseminated via national and international conferences, social media outlets to lay and scientific communities and manuscripts submitted to peer‐reviewed journals for publication.

### 2.10 Training, Quality Assurance and Data Monitoring

All researchers involved in data collection have received training to ensure consistency in assessment procedures. The training sessions covered the use of assessment equipment by discipline specialists (e.g., hearing by HG, vision by MT), administration of questionnaires, use of the Qualtrics system, skills for communicating with participants with hearing and/or vision and cognitive impairments, data cleansing and data checking. Additionally, all researchers underwent MoCA training and certification (https://mocacognition.com/training-certification/). An assessment protocol manual has been provided to all data collectors, providing step‐by‐step instructions. Throughout the study, data will be exported from the Qualtrics platform, saved in a secure repository and screened for any problematic entries or inconsistent responses. Identified issues will be fed back to data collectors to facilitate timely correction and maintain data quality.

### 2.11 Patient and Public Involvement

This study has been shaped by input from older Australians receiving home care services and stakeholders across the aged care and sensory health sectors. Initial feedback was obtained from 11 older adults who reported that hearing and vision impairments had a substantial impact on their daily activities and social participation, alongside unmet care needs, and uncertainty about available intervention options. A Patient and Public Involvement (PPI) advisory group comprising individuals with lived experience of dementia and hearing and/or vision loss has been established and meets every 6 months to provide ongoing input into the design and conduct of the study. In addition, a stakeholder steering group, including policy makers, industry representatives, home care providers (e.g., Ballycara, Bolton Clarke, Brightwater) and hearing, vision and dementia care organizations (e.g., Hearing Australia, Deafness Forum of Australia, Starkey Hearing, Vision Australia, Guide Dogs Australia, Vision 2020, Optometry Australia, Macular Disease Foundation Australia and Dementia Australia), has been consulted through annual meetings to guide the development and implementation of the study. Two meetings have been held to date.

## 3 Discussion

This study addresses an important gap in the evidence base regarding the hearing, vision and cognitive health of older Australians receiving home care services. While the prevalence of hearing, vision and cognitive impairment has been studied in community‐dwelling and residential aged care cohorts [8, 11, 12, 13, 54], little is known about the strategically important home care population in Australia.

Older adults are a rapidly growing sector of the Australian population, projected to increase from 16% in 2020 to approximately 21% to 23% by 2066 [55]. The demand for home care services is therefore expected to expand, driven by population ageing and consumer preference for remaining at home. Supporting older Australians to live independently in their own homes is a key goal of the Australian Government, as reflected in aged care reforms and the Aged Care Act 2024 [56]. Home care is also more cost‐effective than residential aged care, with 2023–2024 government expenditure at AU$11.5 billion for home care compared with AU$21.5 billion for residential aged care, and per‐person expenditure at AU$2349 versus AU$4634, respectively [57].

Data on the hearing, vision and cognitive status of home care users, and the impact of hearing, vision and cognitive impairment on quality of life, functional status and mental well‐being, are essential for guiding health and home care service innovation, workforce training and aged care policy development. However, hearing, vision and cognitive impairment may be especially underrecognized by older adults and their informal caregivers in homecare setting due to other health concerns taking precedence over hearing and vision difficulties, reduced insight into hearing/vision difficulties among those with cognitive difficulties and challenges with seeking help for hearing/vision problems. Mobility limitations and non‐driving status also restrict access to community‐based hearing/vision care. Ageist assumptions may discourage referral or treatment for treatable conditions (e.g., ‘hearing/vision problems are normal for that age’). Failure to detect and treat hearing/vision impairments has significant consequences, including reduced health‐related quality of life [58], increased risk of falls [59] and increased likelihood of transition into residential aged care [5, 60].

Improved recognition of sensory and cognitive impairments can guide the development of individualized care plans that reflect the functional abilities and needs of older adults. Tailored care could include optimizing hearing and vision, supporting device use (e.g., hearing aids, glasses, etc.), adapting communication approaches, environmental modifications, fall prevention strategies and social support initiatives to counteract isolation [61].

By understanding the prevalence of underdiagnosed sensory and cognitive impairments, this study may contribute to the development of assessment protocols and workforce training initiatives aimed at improving the early detection and management of hearing, vision and cognitive impairments in home care settings. Low‐cost portable objective assessments, such as those employed in this study (e.g., HearX HearTest, PEEK Acuity App, SPARCS), could be suitable for use by aged care providers and facilitate coordination of care between home care providers, audiologists, optometrists and specialist services [30]. Embedding hearing and vision assessments into aged care aligns with recommendations from the final report of the Royal Commission into Aged Care Quality and Safety, which called for improved access, equity and person‐centred care that supports independence and dignity [62]. This would entail home care recipients receiving appropriate hearing/vision interventions, including hearing aids, glasses, cognitive support, or multi‐modal rehabilitation.

This protocol has several strengths. First, the use of validated, objective measures of hearing, vision and cognition ensures robust estimates of impairment that avoid the biases and under‐identification of self‐reported measures. Second, the multi‐disciplinary research team, including expertise in audiology, optometry, psychology, geriatrics and health economics, reflects the integrated care approach required to address these complex, comorbid conditions. Third, the inclusion of outcomes, including quality of life, functional ability and psychological well‐being, ensures relevance to care planning and evaluation. Finally, the use of portable, inexpensive assessment technologies enhances the potential for future implementation in routine aged care practice. Findings are therefore not only of academic interest but also of relevance to healthcare managers and policymakers seeking to optimize quality of care. While the sample size is adequately powered to estimate levels of impairment, generalizability may be constrained by the under‐representation of individuals from rural and remote communities in Australia.

## 4 Conclusion

This study will provide the first estimates of the prevalence and impact of hearing, vision and cognitive impairments among older Australians receiving home care services. Findings will have implications for healthcare service delivery, informing integration of sensory and cognitive assessment into routine aged care needs assessments and care planning. Findings will also inform aged care staff training and the development of aged care standards and funding models that include consideration of comorbid sensory and cognitive impairments. A better understanding of the sensory and cognitive needs of the important home care population has the potential to reduce care costs and support older people living in their own homes, as well as improve the quality of life and healthy ageing.

## Author Contributions

**Melinda Toomey:** methodology, resources, investigation, project administration, writing – original draft. **Lana Wilson:** methodology, investigation, writing – original draft. **Helen Gurteen:** methodology, investigation, writing – review and editing. **Bronwyn Franco:** Methodology, Investigation, writing – review and editing. **Rebecca Bennett:** funding acquisition, Methodology, writing – review and editing.**Dayna Cenin:** methodology, investigation, writing – review and editing. **Najwan El-Saifi:** methodology, writing – review and editing. **Melanie Ferguson:** funding acquisition, methodology, writing – review and editing. **Yuanyuan Gu:** funding acquisition, methodology, writing – review and editing. **Chyrisse Heine:** funding acquisition, methodology, writing – review and editing. **Lisa Keay:** funding acquisition, methodology, writing – review and editing. **Sheela Kumaran:** funding acquisition, methodology, writing – review and editing. **Sabrina Lenzen:** funding acquisition, methodology, writing – review and editing. **Iracema Leroi:** funding acquisition, methodology, writing – review and editing. **Judy A. Lowthian:** funding acquisition, methodology, writing – review and editing. **Carly Meyer:** funding acquisition, methodology, writing – review and editing. **Leander Mitchell:** funding acquisition, methodology, writing – review and editing. **John Newall:** funding acquisition, methodology, writing – review and editing. **Nancy A. Pachana:** funding acquisition, methodology, writing – review and editing. **Marianne Piano:** funding acquisition, methodology, writing – review and editing. **Smriti Raichand:** methodology, writing – review and editing. **Emma Scanlan:** funding acquisition, methodology, writing – review and editing. **Hamid Sohrab:** funding acquisition, methodology, writing – review and editing. **Piers Dawes:** conceptualization, funding acquisition, methodology, writing – review and editing.

## Ethics Statement

Ethical approval for this study was obtained from The University of Queensland Human Research Ethics Committee (HE002236).

## Consent

Written informed consent was obtained from all participants before data collection. For individuals with cognitive impairment, proxy consent will be provided by a legal guardian, with assent from the participant where possible.

## Conflicts of Interest

The authors declare no conflicts of interest.

## Supporting information

Supplementary_materials.

## Data Availability

Non‐identifiable data generated from this study will be archived in The University of Queensland's Research Data Management System (UQRDM), a publicly accessible repository, in accordance with the National Health and Medical Research Council's Open Access Policy and FAIR Principles.
