# Supplementary material for: Prevalence of Hearing, Vision and Cognitive Impairment and Impact in Older Adults in Home Care: A Study Protocol
Source: Health Expect. 2026 Apr 15;29(2):e70613. doi: 10.1111/hex.70613 (PMC13080888; doi:10.1111/hex.70613)
Supplement: Supplementary file 1 — Supplementary_materials. [file HEX-29-e70613-s001.docx]

Supplement 1 – Assessment result sheet

STROBE Statement—checklist of items that should be included in reports of observational studies

|  | **Item No** | **Recommendation** | **Page  No** |
| --- | --- | --- | --- |
| **Title and abstract** | 1 | (*a*) Indicate the study’s design with a commonly used term in the title or the abstract | 1 |
|  |  | (*b*) Provide in the abstract an informative and balanced summary of what was done and what was found | 3 |
| **Introduction** | | | |
| Background/rationale | 2 | Explain the scientific background and rationale for the investigation being reported | 4-6 |
| Objectives | 3 | State specific objectives, including any prespecified hypotheses | 6 |
| **Methods** | | | |
| Study design | 4 | Present key elements of study design early in the paper | 6 |
| Setting | 5 | Describe the setting, locations, and relevant dates, including periods of recruitment, exposure, follow-up, and data collection | 6-8 |
| Participants | 6 | (*a*) *Cohort study*—Give the eligibility criteria, and the sources and methods of selection of participants. Describe methods of follow-up  *Case-control study*—Give the eligibility criteria, and the sources and methods of case ascertainment and control selection. Give the rationale for the choice of cases and controls  *Cross-sectional study*—Give the eligibility criteria, and the sources and methods of selection of participants | 7 |
|  |  | (*b*) *Cohort study*—For matched studies, give matching criteria and number of exposed and unexposed  *Case-control study*—For matched studies, give matching criteria and the number of controls per case | N/A |
| Variables | 7 | Clearly define all outcomes, exposures, predictors, potential confounders, and effect modifiers. Give diagnostic criteria, if applicable | 9-16 |
| Data sources/ measurement | 8* | For each variable of interest, give sources of data and details of methods of assessment (measurement). Describe comparability of assessment methods if there is more than one group | *9-16* |
| Bias | 9 | Describe any efforts to address potential sources of bias | NR |
| Study size | 10 | Explain how the study size was arrived at | 7 |
| Quantitative variables | 11 | Explain how quantitative variables were handled in the analyses. If applicable, describe which groupings were chosen and why | 7, 17 |
| Statistical methods | 12 | (*a*) Describe all statistical methods, including those used to control for confounding | 17 |
|  |  | (*b*) Describe any methods used to examine subgroups and interactions | 17 |
|  |  | (*c*) Explain how missing data were addressed |  |
|  |  | (*d*) *Cohort study*—If applicable, explain how loss to follow-up was addressed  *Case-control study*—If applicable, explain how matching of cases and controls was addressed  *Cross-sectional study*—If applicable, describe analytical methods taking account of sampling strategy | NR |
|  |  | (*e*) Describe any sensitivity analyses | NR |

N/A not applicable, NR not reported.

Supplement 2 – Assessment result sheet


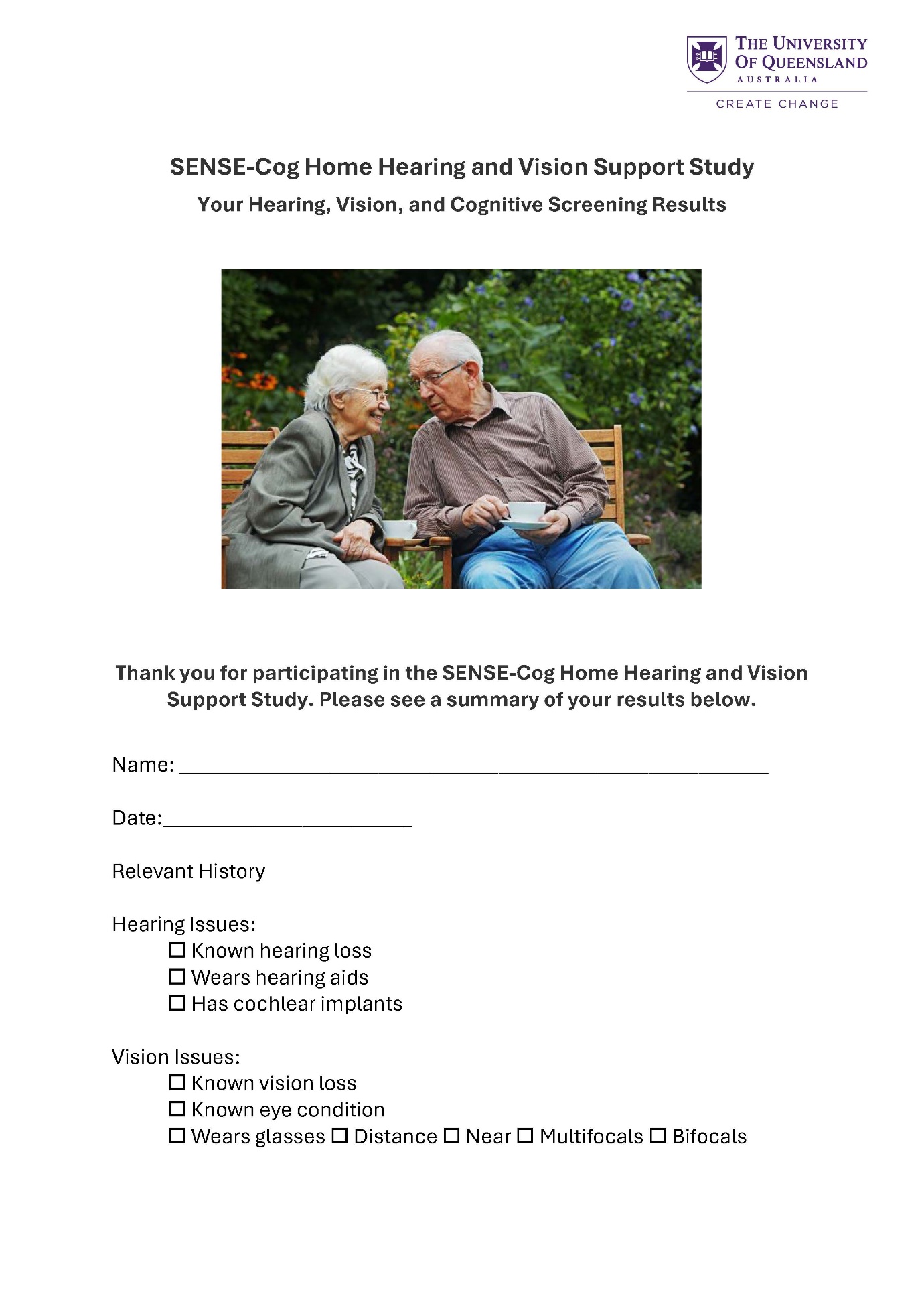


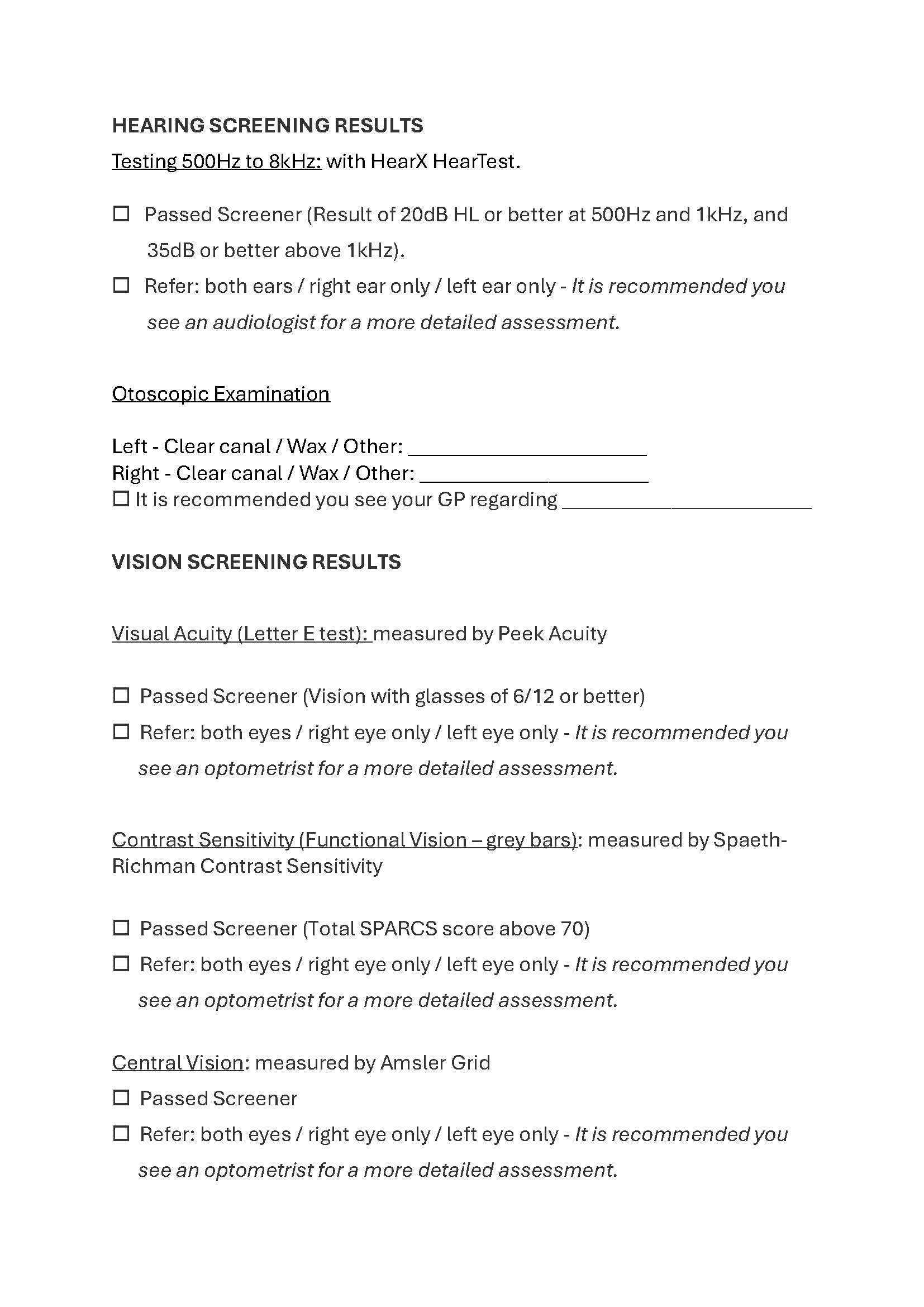


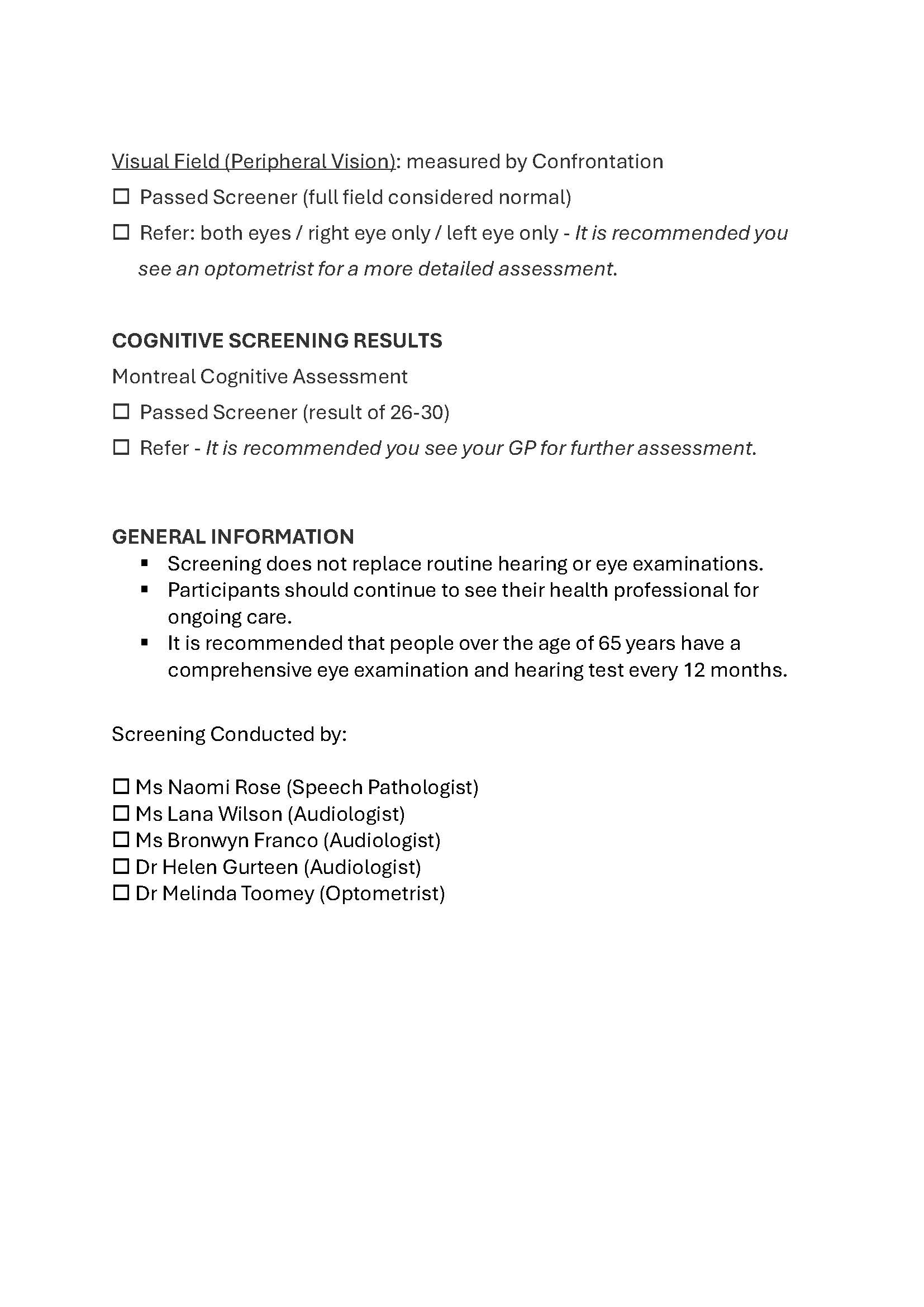


Supplement 3 – Sociodemographic Questionnaire

Q1. I live at home, am 65 years or older and receive home care services

- Yes, please proceed to question 2
- No, Please STOP. This form is not suitable to capture your responses

**Please answer these questions about yourself**

Q2. What is your age? _________ years

Q3. What is your gender?

- Male
- Female
- Other
- Prefer not to say

Q4. Do you identify as an Aboriginal and/or Torres Strait Islander person?

- Yes
- No
- refer not to say

Q5. Were you born overseas?

- Yes – proceed to the next question
- No – proceed to question 8
- Prefer not to say – proceed to question 8

Q6. In what country were you born? ___________________

Q7. How long have you lived in Australia?

- Less than 1 year
- 1 – 5 years
- 5 – 10 years
- More than 10 years

Q8. Do you speak a language other than English at home?

- Yes (please list language/s): _______________________
- No
- Prefer not to say

Q9. What is your marital status?

- Married
- Widowed
- Divorced
- Separated
- Single
- Prefer not to say

Q10. What is your highest level of education?

- Primary school education
- Secondary school education
- Diploma, Certificate or equivalent
- Bachelor’s degree or equivalent
- Postgraduate qualifications (Masters or Doctoral degree)
- Other qualifications (please provide details): ________________________________

Q11. What is your current or former occupation? _________________

Q12. What is your postcode? __________

Q13. How long have you received home care services?

- Less than 1 year
- 1 – 2 years
- More than 2 years

Q14. What home care package level do you receive?

- Level 1
- Level 2
- Level 3
- Level 4

Q15. What services do you receive in your home care package? (Tick all that apply)

- Personal care (e.g., bathing, toileting, dressing and other daily activities)
- Clinical care (e.g., general and specialised nursing and other health care services)
- Allied health services (e.g., occupational therapy, physiotherapy, mental health services etc))
- Transport and outings
- Socialising and companionship (e.g., community activities, hobbies and lifestyle interests)
- Cleaning and household tasks
- Gardening
- In-home respite
- Meal preparation
- Mobility aids and others
- Other (please specify ____________________)

**Health Status**

Q16. How would you describe your general health status?

- In good physical health (no illness or disabilities)
- Mildly physically impaired (minor illness or disabilities)
- Moderately physically impaired (requires substantial treatment)
- Severely physically impaired (requires extensive treatment)
- Totally physically impaired (confined to bed)

Q17. Do you have any of the following chronic health conditions (tick all that apply)

- Asthma
- Arthritis (e.g., osteoarthritis, rheumatoid arthritis)
- Cancer
- Chronic kidney disease
- Chronic obstructive pulmonary disease
- Coronary heart disease
- Diabetes
- High blood pressure
- Osteoporosis
- Stroke
- Other (please specify) ___________________________

Q18. Do you take any medications or supplements? Yes/No

Q19. If yes, please list the medications you take: _______________________

Q20. How often do you visit your general practitioner?

- Once a week
- Once a month
- Once every 3 months
- Once every 6 months
- Once every year
- Other (please specify) ____________________________

**Hearing Function**

Q21. Do you feel you have a hearing loss?

- Yes
- No
- Don’t know

Q22. About how long have you experienced hearing difficulties?

- Less than 1 year
- 1 – 5 years
- 5 – 10 years
- More than 10 years
- Don’t know

Q23. Have you ever seen a hearing health care professional (audiologist) about hearing problems?

- Yes
- No
- Don’t know

Q24 (If yes) Have you had a diagnosis of hearing loss?

- Yes
- No
- Don’t know

Q25. (If yes) Do you use a hearing aid or cochlear implant?

- Yes – right ear
- Yes – left ear
- Yes, both ears
- No
- Don’t know

Q26. (If yes) Think about how much you used your present hearing aid(s) over the past two weeks. On an average day, how many hours did you use the hearing aid(s)?

- None
- Less than 1 hour a day
- 1 to 4 hours a day
- 4 to 8 hours a day
- More than 8 hours a day

**Vision Function**

Q27. Do you use glasses?

- Yes
- No
- Don’t know

Q28. Do you have difficulty seeing even when wearing glasses (if you own them)?

- Yes
- No
- Don’t know

Q29. (If yes, About how long have you had difficulty seeing even when wearing glasses?

- Less than 1 year
- 1 – 5 years
- 5 – 10 years
- More than 10 years
- Don’t know

Q30. Have you ever seen a vision health care professional (optometrist, ophthalmologist) about your vision problems?

- Yes
- No
- Don’t know

Q31. (If yes) Have you had a diagnosis of vision loss?

- Yes – age-related macular degeneration
- Yes – glaucoma
- Yes – cataracts
- Yes - other (please specify) ______________
- No
- Don’t know

Q32. (If yes to cataracts) Have you previously had cataract surgery?

- Yes, one eye
- Yes, both eyes
- No
- Don’t know

Q33. (If yes to cataracts) Are you scheduled to have cataract surgery in the next 6 months?

- Yes
- No
- Don’t know

Q34. (If yes to age-related macular degeneration) For your macular degeneration, do you visit your ophthalmologist regularly for injections into your eye to treat the condition?

- Yes
- No
- Don’t know

Q35. Do you use glasses or low vision devices?

- Yes, for seeing distance and near
- Yes, only for near work / reading
- Yes, only for distance
- No
- Don’t know

**Memory Function**

Q36. Do you have problems with your memory?

- Yes
- No
- Don’t know

Q37. (If yes) Have you had a diagnosis of dementia?

- Yes
- No
- Don’t know

Q38. (If yes) About how long have you had a diagnosis of dementia?

- Less than 1 year
- 1 – 5 years
- 5 – 10 years
- More than 10 years
- Don’t know

Q39. (If yes) What type of dementia?

- Alzheimer’s
- Vascular
- Fronto-Temporal
- Lewy bodies
- Mixed Dementia
- Parkinson’s
- Dementia not otherwise specified
- Other
- Don’t know

**THANK YOU VERY MUCH FOR COMPLETING THIS QUESTIONNAIRE**
